# Unique Clinical, Immune, and Genetic Signature in Patients with Borrelial Meningoradiculoneuritis^1^

**DOI:** 10.3201/eid2804.211831

**Published:** 2022-04

**Authors:** Katarina Ogrinc, Sergio A. Hernández, Miša Korva, Petra Bogovič, Tereza Rojko, Lara Lusa, Geena Chiumento, Franc Strle, Klemen Strle

**Affiliations:** University Medical Center Ljubljana, Ljubljana, Slovenia (K. Ogrinc, P. Bogovič, T. Rojko, F. Strle);; New York State Department of Health, Albany, New York, USA (S.A. Hernández, K. Strle);; University of Ljubljana, Ljubljana (M. Korva, L. Lusa);; Massachusetts Department of Public Health, Boston, Massachusetts, USA (G. Chiumento)

**Keywords:** Lyme disease, neuroborreliosis, meningoradiculoneuritis, peripheral facial palsy, meningitis/encephalitis, immune response, inflammation, cytokines, genetics, polymorphism, vector-borne infections, bacteria, Slovenia, United States

## Abstract

Lyme neuroborreliosis (LNB) in Europe may manifest with painful meningoradiculoneuritis (also known as Bannwarth syndrome) or lymphocytic meningitis with or without cranial neuritis (peripheral facial palsy). We assessed host immune responses and the prevalence of TLR1 (toll-like receptor 1)–1805GG polymorphism to gain insights into the pathophysiology of these conditions. Regardless of LNB manifestation, most mediators associated with innate and adaptive immune responses were concentrated in cerebrospinal fluid; serum levels were unremarkable. When stratified by specific clinical manifestation, patients with meningoradiculoneuritis had higher levels of B-cell chemoattractants CXC motif chemokine ligand (CXCL) 12 and CXCL13 and T-cell–associated mediators CXCL9, CXCL10, and interleukin 17, compared with those without radicular pain. Moreover, these patients had a higher frequency of TLR1–1805GG polymorphism and more constitutional symptoms. These findings demonstrate that meningoradiculoneuritis is a distinct clinical entity with unique immune and genetic pathophysiology, providing new considerations for the study of LNB and borrelial meningoradiculitis.

## Abstract 2

This form of Lyme neuroborreliosis is a distinct clinical entity with specific signs and symptoms.

Lyme borreliosis, which is caused by several species of the *Borrelia burgdorferi* sensu lato complex, is the most common vectorborne disease in the Northern Hemisphere. The first sign of infection is usually an expanding skin lesion, erythema migrans (EM), that appears within days or weeks at the site of the tick bite. If untreated, the infection may disseminate to involve other organ systems, including the central nervous system (CNS), heart, or joints (*1*,*2*).

Lyme neuroborreliosis (LNB) is the most common extracutaneous manifestation of Lyme borreliosis in Europe and the second most common such manifestation in North America (*1*,*2*). In Europe, LNB in adult patients typically begins as painful meningoradiculoneuritis (also known as Garin-Bujadoux-Bannwarth syndrome or Bannwarth syndrome), which is rare in the United States, or with cranial neuritis characterized by peripheral facial palsy (PFP) or lymphocytic meningitis (*1*–*4*). In addition, a subset of patients experience EM and symptoms indicative of LNB (e.g., headache, vertigo, concentration disturbance), but do not fulfill the European diagnostic criteria for LNB, namely cerebrospinal fluid (CSF) pleocytosis and intrathecal borrelial antibody production (*5*,*6*). This subset poses a particular diagnostic and clinical challenge because the etiology of illness is often unclear.

Pathogenic mechanisms that account for different clinical manifestations of LNB are not well understood. Clinical heterogeneity in Lyme borreliosis has been largely attributed to differences in the infecting *B. burgdorferi* s.l. species (*1*,*2*). In Europe, LNB is caused predominantly by *B. garinii* (and closely related *B. bavariensis*) but only rarely by *B. afzelii*, which is associated with skin manifestations, or *B. burgdorferi* sensu stricto, which is highly arthritogenic, implying a strong species-specific imprinting of clinically evidenced disease (*1*,*2*,*7*,*8*). However, although *B. garinii* has particular proclivity for the central nervous system (CNS) and undoubtedly serves as the initial trigger of LNB, Lyme borreliae do not express any known toxins that cause disease. Rather, the host immune response, which is shaped by various *Borrelia* species (*9*–*13*), is thought to be a key determinant of the clinical signs and symptoms of Lyme borreliosis, including LNB.

In addition to infection with a particular *Borrelia* species or strain, the immune responses are further augmented by alterations in the human genome. We have previously demonstrated that a single-nucleotide polymorphism (SNP; 1805GG) in the host toll-like receptor 1 (TLR1), a key pathogen sensing receptor for borrelia, is associated with more symptomatic early Lyme borreliosis and a greater frequency of postantibiotic, chronic inflammatory Lyme arthritis (*12*,*14*). This TLR1–1805GG SNP is associated with severe disease presumably because it enables maladaptive immune responses; EM patients with the TLR1–1805GG SNP had substantially higher levels of pro-inflammatory cytokines and chemokines in blood compared with those without the SNP. Moreover, in patients with Lyme arthritis, this SNP was associated with exceptionally high levels of the interferon (IFN) γ and IFN-γ–associated mediators CXC motif chemokine ligand (CXCL) 9 and CXCL10 in synovial fluid, supporting the predominant cellular and inflammatory T-helper 1 (Th1) responses in joints in these patients. Thus, host immune response, which is shaped by both microbial and host genetics, is a key driver of the clinical heterogeneity in Lyme borreliosis in general and probably in LNB, although this has not been tested directly.

Several studies in Europe and the United States have characterized host immune responses in patients with LNB in an effort to gain insights into pathogenesis and to identify biomarkers to aid in diagnosis (*15*–*18*). These studies demonstrated the activation of a broad-ranging innate and adaptive immune response in patients with LNB. In particular, LNB patients have marked CSF levels of CXCL13, a B-cell chemoattractant, compared with patients with other neurologic conditions (*15*–*19*), prompting the use of CXCL13 as an adjunct diagnostic biomarker of LNB (*19*–*26*). However, the role of host immunity in the pathogenesis of clinically distinct LNB manifestations and the possibility that these outcomes are associated with specific host genetic predisposition has not been explored.

We investigated the role of innate and adaptive immune responses and the TLR1–1805GG SNP in specific clinical manifestations of LNB. Our findings demonstrate that borrelial meningoradiculoneuritis is a distinct clinical entity with unique immune and genetic pathophysiology and offer new insights into the pathogenesis and diagnosis of this condition.

## Methods

### Patient Selection

This study is based on 61 adult patients who met the modified European diagnostic criteria for LNB (*5*), defined by the presence of symptoms suggestive of neurologic involvement; CSF lymphocytic pleocytosis; demonstration of borrelial infection with intrathecal synthesis of borrelia antibodies (IT-Bb-Abs), isolation of borreliae from CSF; or presence of EM. For comparison, we included 59 patients with signs and symptoms indicative of LNB but without pleocytosis and termed them suspected LNB. All patients were seen at the Lyme borreliosis outpatient clinic by the same clinical team in the Department of Infectious Diseases, University Medical Center Ljubljana, Ljubljana, Slovenia, during 2006–2013. The Medical Ethics Committee of the Ministry of Health, Republic of Slovenia (no. 35/08/06), and the Institutional Review Board of the New York Department of Health (IRB protocol no. 20-013) approved the study.

### Clinical Evaluation

We collected clinical and demographic information using a structured questionnaire as described previously (*3*). In addition to their general medical history, patients were asked about tick bites, EM, and new onset or worsening of symptoms. We also assessed signs indicative of neurologic involvement (meningeal signs, PFP, other cranial nerve palsy, sensory or motor deficits, tremor) and the presence of EM (*27*). We subsequently grouped patients into 3 categories according to clinical and diagnostic criteria: 1) painful meningoradiculoneuritis accompanied by CSF pleocytosis; 2) LNB without radicular pain, consisting of PFP with CSF pleocytosis or lymphocytic meningitis without cranial neuritis (PFP/meningitis); and 3) suspected LNB with EM and neurologic symptoms suggestive of LNB, but normal CSF cell counts (*3*,*6*). Serum and CSF samples were obtained at first visit, usually before antimicrobial therapy, and stored at −80°C for subsequent analyses.

### Laboratory Evaluation

We defined CSF pleocytosis as a leukocyte count >5 × 10^6^ cells/L. We determined IgM and IgG levels against *B. burgdorferi* s.l. in serum and CSF with indirect chemiluminescence immunoassay using recombinant OspC and VlsE antigens (LIAISON; Diasorin, https://www.diasorin.com). We assessed intrathecal synthesis of *Borrelia* IgG as described previously (*28*); antibody index values >1.4 were indicative of IT-Abs against *B. burgdorferi* s.l. We cultivated *Borrelia* in CSF as reported previously (*29*–*31*).

### Cytokine and Chemokine Determinations

We assessed the levels of 17 mediators associated with innate (CC motif chemokine ligand [CCL] 2, CCL3, CCL4, interleukin [IL] 8, IL-10, tumor necrosis factor, IFN-α), and adaptive T-cell (T_H_1, IFN-γ, CXCL9, CXCL10; T_H_17, IL-17A, IL-21, IL-23; CCL19, CCL21) or B-cell (CXCL12, CXCL13) immune responses. We tested for these levels in matched patient serum and CSF samples using bead-based multiplex assays (EMD Millipore, https://www.emdmillipore.com) coupled with the Luminex-200 Analyzer (Luminex, https://www.luminexcorp.com). We quantified each inflammatory mediator in all samples in one complete experiment to minimize sample freeze-thaw and assay variation. We then stratified cytokine and chemokine levels according to clinical and laboratory findings in the same patients.

### TLR1–1805 Genotyping

We determined TLR1–1805 genotypes (TT, TG, GG; G denotes the SNP) using PCR amplification followed by restriction fragment length polymorphism (RFLP) as described previously (*12*). In brief, we extracted total DNA from patients’ blood (QIAamp DNA Blood Mini Kit; QIAGEN, https://www.qiagen.com) and amplified it using specific primers: forward: 5′-GCAGGGGACAATCCATTCCAA-3′; reverse: 5′CCCAGAAAGAATCGTGCCCA-3′ (IDT). Each reaction contained 0.4 µM forward/reverse primers, 2 µL DNA (≥50 ng), and 2XDreamTaq PCR Master Mix (ThermoFisher Scientific, https://www.thermofisher.com). PCR amplicons were digested with *Pst*I enzyme (New England BioLabs, https://www.neb.com) and visualized by electrophoresis (1.5% agarose gel, GelRed nucleic acid stain). We determined TLR1–1805 genotype in 65 patients with confirmed LNB (meningoradiculoneuritis, n = 40; LNB without radiculoneuritis [termed PFP/meningitis], n = 25) and for comparison in 71 EM patients without neurologic involvement. Because of the requirement for consent for genetic studies, these 65 LNB patients represent a different cohort from the 61 patients assessed in the rest of the study. In addition, we ascertained TLR1–1805 genotype in the general European population (n = 503) by datamining the Ensembl genome browser 104 (https://useast.ensembl.org/index.html).

### Statistical Analyses

We evaluated categorical variables (presence/absence of symptoms or TLR1-SNP) using Fisher exact test. We assessed quantitative variables (number of symptoms, cytokine levels) using Mann-Whitney nonparametric rank-sum test in Prism version 9.1.1 (https://www.graphpad.com). We applied Spearman correlation for cytokine associations. We adjusted for multiple comparisons using the Benjamini-Hochberg test; we set the false discovery rate (FDR) to 0.05 and considered p<0.05 statistically significant.

## Results

### Clinical Characteristics According to LNB Presentation

**Figure 1:**
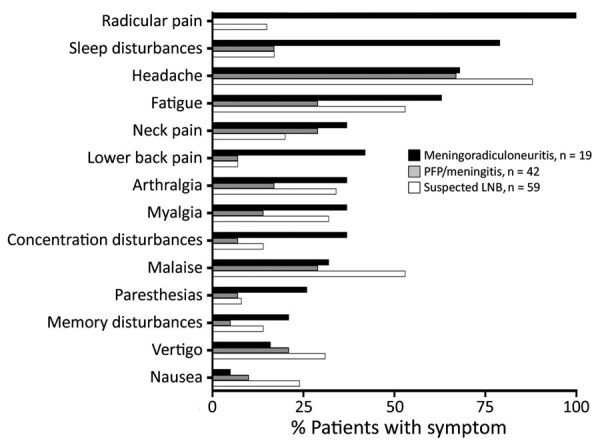
Frequency of individual signs and symptoms at study enrollment among patients with LNB treated in Ljubljana, Slovenia, during 2006–2013. Patients were stratified into 3 groups: meningoradiculoneuritis, PFP/meningitis without radicular pain, or patients with suspected LNB who had EM and signs and symptoms suggestive of LNB but did not meet the standard diagnostic criteria. *Meningoradiculoneuritis vs. PFP/meningitis, p<0.05; †meningoradiculoneuritis vs. suspected LNB, p<0.005. LNB, Lyme neuroborreliosis; PFP, peripheral facial palsy.

Of the 120 patients in this study, 19 had meningoradiculoneuritis, 42 had LNB without radiculoneuritis (PFP/meningitis; 35 PFP, 7 lymphocytic meningitis), and 59 were classified as suspected LNB because they had clinical signs and symptoms indicative of LNB (EM with neurologic symptoms) but lacked CSF pleocytosis (*5*) (Table 1). Seventy-two participants were female (60%) and 48 male (40%); median age was 51 years. When stratified by specific clinical presentation, patients with meningoradiculoneuritis were more likely to have had an EM skin lesion during the course of disease than LNB patients without radicular pain (68% vs. 24%; p = 0.004). Meningoradiculoneuritis patients had more symptoms than LNB patients without radiculitis (median 6 vs. 3 symptoms; p<0.001) and their symptoms lasted longer (30 days vs. 10 days; p<0.001). Meningoradiculoneuritis patients had more symptoms (median 6 vs. 4 symptoms; p = 0.01) and symptoms of longer duration (30 vs. 16 days; p<0.03) than patients with suspected LNB. Of the 13 constitutional symptoms we assessed, patients with meningoradiculoneuritis had a higher frequency of 8 specific symptoms, 4 of which reached statistical significance (Figure 1). The most common symptoms were sleep disturbance, headache, and fatigue.

**Table 1:** Clinical characteristics of patients with confirmed or suspected Lyme neuroborreliosis, Ljubljana, Slovenia, 2006–2013*

Characteristic	MR, n = 19	PFP/meningitis, n = 42	Suspected LNB, n = 59	p value†		
				MR vs. PFP‡	MR vs. sLNB	PFP‡ vs. sLNB
Demographics						
Age, y	51 (21–73)	52 (15–81)	51 (25–83)	0.4	0.8	0.4
Sex						
F	9 (47)	17 (40)	45 (76)	0.8	**0.02**	**0.0004**
M	10 (53)	25 (60)	14 (24)			
No. tick bites/y	1 (0–17)	2 (0–20)	2 (0–30)	0.9	0.7	0.5
Clinical characteristics						
Current or recent EM	13 (68)	10 (24)	59 (100)§	**0.004**	**0.0001**	**<0.0001**
Solitary EM	12 (63)	6 (14)	53 (90)	**0.0007**	**0.01**	**<0.0001**
Multiple EM	1 (5)	4 (10)	6 (10)	1	1	1
EM duration, d	35 (6–128)	11 (3–60)	21 (1–240)	0.2	0.9	**0.01**
No. symptoms/patient	6 (1–12)	3 (1–10)	4 (1–13)	**<0.0001**	**0.01**	**0.001**
Duration of symptoms, d	30 (7–75)	10 (3–365)	16 (2–270)	**0.001**	0.2	**0.03**
Radicular pain	19 (100%)	0 (0%)	9 (15%)	**<0.0001**	**<0.0001**	**0.01**
Peripheral facial palsy	9 (47%)	35 (83%)	0 (0%)	**0.01**	**<0.0001**	**<0.0001**
CSF findings						
Pleocytosis	19 (100)	42 (100)	0	1.0	**<0.0001**	**<0.0001**
Leukocyte count, × 10^6^ cells/L	160 (15–886)	56 (6–1579)	1 (0–4)	**0.005**	**<0.0001**	**<0.0001**
Lymphocyte count no. × 10^6^ cells/L	144 (15–811)	47 (4–1477)	1 (0–4)	**0.005**	**<0.0001**	**<0.0001**
*Borrelia* culture positivity¶	4 (21)	3 (7)	0	0.2	**0.002**	0.06

*Values are no. (%) or median (range). CSF, cerebrospinal fluid; EM, erythema migrans; LNB, Lyme neuroborreliosis; MR, meningoradiculoneuritis; PFP, peripheral facial palsy; sLNB, suspected Lyme neuroborreliosis. †Statistical analyses were performed using Mann-Whitney nonparametric rank sum test. Median values are shown. Bold type indicates statistically significant p values after Benjamini-Hochberg correction for multiple comparisons (false discovery rate = 0.05). ‡PFP group includes 42 LNB patients without radicular pain (35 patients with PFP and 7 with lymphocytic meningitis). §Presence of EM was a criterion for suspected LNB. ¶*Borrelia burgdorferi* sensu lato positive culture result in CSF.

### Immune Responses in Serum and CSF

In 61 patients with confirmed LNB (meningoradiculoneuritis or PFP/meningitis), most cytokines and chemokines associated with adaptive T-cell and B cell immune responses were more highly concentrated in CSF, the site of disease, than in serum (Table 2). This finding was particularly apparent for IFN-γ–inducible chemokines CXCL9 (median 347 vs. 170 pg/mL; p = 0.02) and CXCL10 (median 4,139 vs. 143 pg/mL; p<0.001) which recruit CD4+ T cells, as well as B-cell chemoattractants CXCL12 (median 1,541 vs. 820 pg/mL; p<0.001) and CXCL13 (median 352 vs. 9 pg/mL; p<0.001) which recruit B cells to site of infection. In contrast, mediators associated with innate and Th17 immune responses were present at lower overall concentrations and often at similar levels in serum and CSF (Table 2).

**Table 2:** Immune mediators in cerebrospinal fluid and serum in 61 confirmed patients with Lyme neuroborreliosis, Ljubljana, Slovenia, 2006–2013*

Cytokine or chemokine	CSF, pg/mL	Serum, pg/mL	p value†
Innate			
IL-8	**39** (8–289)	**8** (0–473)	**<0.0001**
IL-10	**0.3** (0–54)	**0.3** (0–28)	**0.0001**
TNF	5 (1–54)	7 (0–170)	0.3
IFN-α	**88** (0–406)	**22** (0–205)	**<0.0001**
CCL2	**343** (71–10,000)	**213** (7–516)	**<0.0001**
CCL3	5 (0.3–16)	1 (0.3–227)	0.07
CCL4	**5** (0.3–139)	**20** (0.3–221)	**<0.0001**
T-cell adaptive			
IFNγ	2 (0–77)	1 (0–25)	0.3
CXCL9	**347** (4–6833)	**170** (2–3,508)	**0.02**
CXCL10	**4,139** (156–46,025)	**143** (3–932)	**<0.0001**
IL-17	**3** (0–8)	**2** (0–25)	**0.02**
IL-23	**2** (2–33)	**2** (2–4,949)	**0.01**
GM-CSF	**5** (0–22)	**3** (0–22)	**<0.0001**
CCL19	**84** (19–768)	**28** (2–143)	**<0.0001**
CCL21	17 (17–241)	17 (17–541)	0.7
B-cell adaptive			
CXCL12	**1,541** (89–5,518)	**820** (89–4184)	**<0.0001**
CXCL13	**352** (1–76,869)	**9** (1–66)	**<0.0001**

*Values are median (range). CCL, CC motif chemokine ligand; CSF, cerebrospinal fluid; CXCL, CXC motif chemokine ligand; IL, interleukin; IFN, interferon; TNF, tumor necrosis factor. †Statistical analyses were performed using Mann-Whitney non-parametric rank sum test. Bold type indicates statistically significant p values after Benjamini-Hochberg correction for multiple comparisons (false discovery rate FDR = 0.05).

### Immune Responses According to LNB Presentation

**Figure 2:**
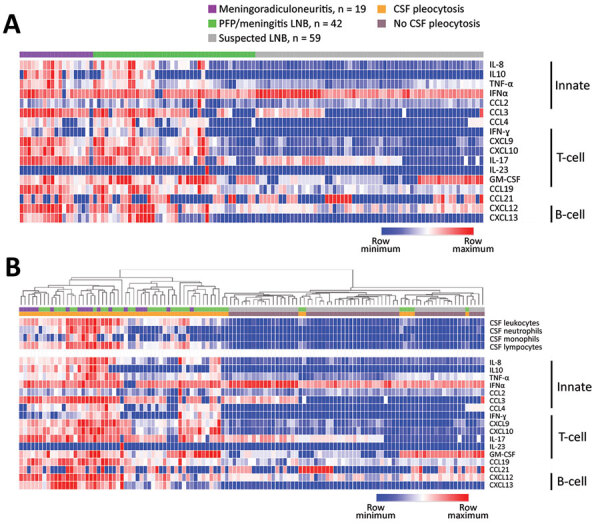
Heat map analysis of inflammatory mediators in CSF stratified by clinical presentation of LNB among patients with Lyme neuroborreliosis treated in Ljubljana, Slovenia, during 2006–2013. A) Levels of inflammatory mediators stratified by LNB manifestation. B) Hierarchical clustering analysis (Euclidian distance) of inflammatory mediators and CSF leukocyte counts. We assessed the levels of 17 cytokines and chemokines associated with innate and adaptive (T- and B-cell) responses in CSF of patients with meningoradiculoneuritis, PFP/meningitis, or suspected LNB. Each column represents an individual patient, with the corresponding mediators along the different rows. Heat map was constructed using Morpheus software (https://software.broadinstitute.org/morpheus). In the case of IFN-α, 3 values were exceptionally low (at lower limit of detection), resulting in disproportionately intense red coloring of other values. CSF, cerebrospinal fluid; CXCL, CXC motif chemokine ligand; GM-CSF, granulocyte macrophage colony–stimulating factor; IL, interleukin; IFN, interferon; LNB, Lyme neuroborreliosis; MIP, macrophage inflammatory protein; PFP, peripheral facial palsy; TNF, tumor necrosis factor.

To determine if patients with different clinical presentations of LNB carry distinct immune signatures, we stratified cytokine and chemokine levels in CSF into 3 groups: meningoradiculoneuritis (n = 19), PFP/meningitis (n = 42), and suspected LNB (n = 59). Heat map analyses of global immune responses revealed that patients with meningoradiculoneuritis have the highest levels of most mediators tested; those with PFP/meningitis have intermediate levels, and those with suspected LNB have the lowest levels (Figure 2, panel A). We obtained similar results using unguided hierarchical clustering (Figure 2, panel B). Patients with meningoradiculoneuritis clustered primarily with the highest cytokine and chemokine levels and largest CSF leukocyte counts, whereas those with suspected LNB clustered with the lowest cytokine and chemokine levels and CSF leukocyte counts. Patients with PFP/meningitis were more interspersed. We observed these trends for cytokines and chemokines associated with both innate and adaptive immune responses.

**Figure 3:**
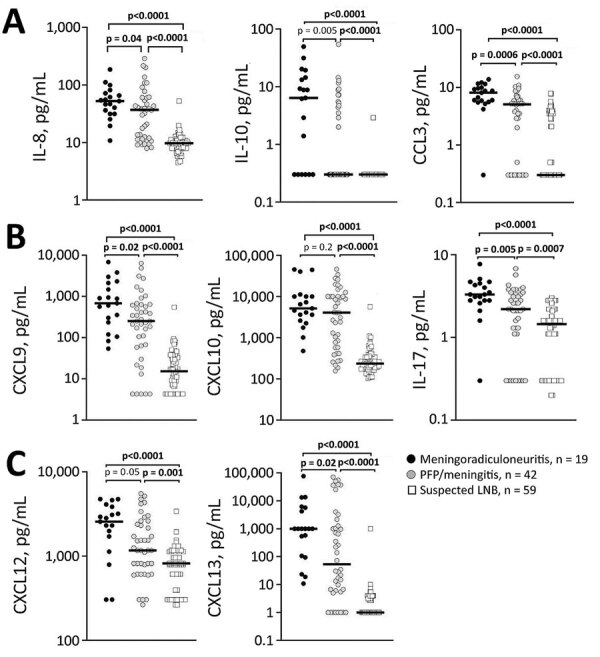
Statistical correlation of the levels of 8 immune mediators in CSF, by clinical manifestation, among patients with LNB treated in Ljubljana, Slovenia, during 2006–2013. A) Mediators associated with innate response are IL-8, IL-10, and CCL3. B) Mediators associated with T-cell adaptive response are CXCL9, CXCL10, and IL-18. C) Mediators associated with B-cell adaptive response are CXCL12 and CXCL13. Black circles represent meningoradiculoneuritis (n = 19); white circles, PFP/meningitis (n = 42); and white squares, suspected LNB (n = 59). Horizontal lines represent median values. Statistical analyses were performed using nonparametric Mann-Whitney rank sum test. p values indicate largest differences for individual patients among groups. CCL, CC motif chemokine ligand; CSF, cerebrospinal fluid; CXCL, CXC motif chemokine ligand; IL, interleukin; LNB, Lyme neuroborreliosis; PFP, peripheral facial palsy.

**Figure 4:**
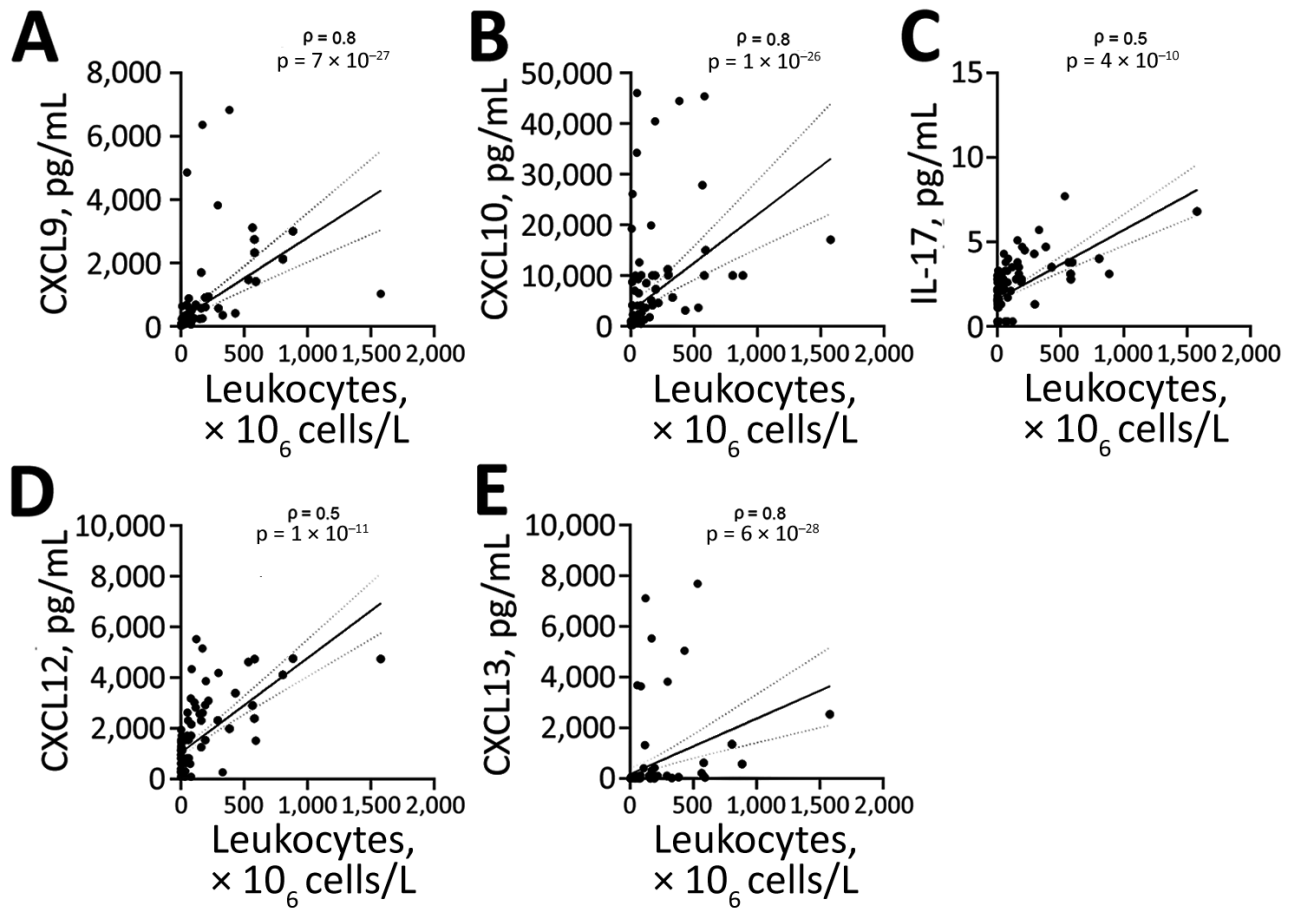
Correlation between the levels of 5 inflammatory mediators in CSF and CSF leukocyte counts among 120 patients with Lyme neuroborreliosis treated in Ljubljana, Slovenia, during 2006–2013. A) CXCL9; B) CXCL10; C) IL-17; D) CXCL12; E) CXCL13. Solid lines inside the graph represent linear regression; dotted lines indicate 95% CIs. ρ and p values were derived using the Spearman correlation. CSF, cerebrospinal fluid.

We identified through statistical analyses 8 cytokines and chemokines with significant differences between groups (Figure 3), including mediators associated with innate (IL-8, IL-10, CCL3), and adaptive T-cell (CXCL9, CXCL10, IL-17) and B-cell (CXCL12, CXCL13) immune responses. We observed the highest levels and most pronounced differences between groups for B-cell chemoattractants CXCL12 (median values for meningoradiculoneuritis groups, 2,568 pg/mL; for PFP/meningitis, 1,170 pg/mL; for suspected LNB, 819 pg/mL) and CXCL13 (meningoradiculoneuritis, 1,000 pg/mL; PFP/meningitis, 53 pg/mL; suspected LNB, 1 pg/mL) and for T-cell chemoattractants CXCL9 (meningoradiculoneuritis, 680 pg/mL; PFP/meningitis, 252 pg/mL; suspected LNB 15 pg/mL) and CXCL10 (meningoradiculoneuritis, 5,159 pg/mL; PFP/meningitis, 4,087 pg/mL; suspected LNB, 236 pg/mL). These mediators correlated strongly with leukocyte counts in CSF (p<0.001) (Figure 4), implying the predominance of CSF-localized T- and B-cell responses in meningoradiculoneuritis.

### Frequency of TLR1–1805GG Polymorphism in LNB

**Figure 5:**
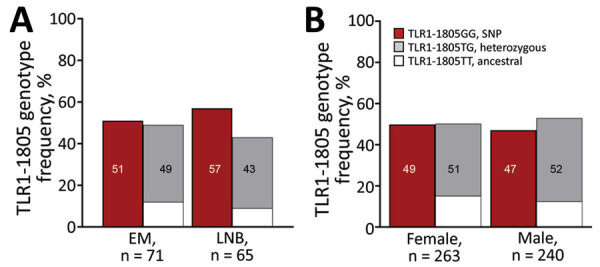
Frequency of TLR1–1805GG polymorphism among patients with Lyme borreliosis treated in Ljubljana, Slovenia, during 2006–2013 (A), compared with the general population of Europe (B). A) Lyme borreliosis patients with EM vs. LNB; B) general population by female vs. male sex. The TLR1 SNP results in an exchange of thymine (T, ancestral) with a guanine (G) allele at position 1805; GG corresponds to both copies of the SNP allele (SNP), TG with one copy (heterozygous), and TT with no copies (ancestral). The information in the general population was obtained from Ensemble genome browser 104 and is based on data from the 1,000 Genomes Project (https://useast.ensembl.org/index.html). EM, erythema migrans; LNB, Lyme neuroborreliosis; SNP, single-nucleotide polymorphism.

**Figure 6:**
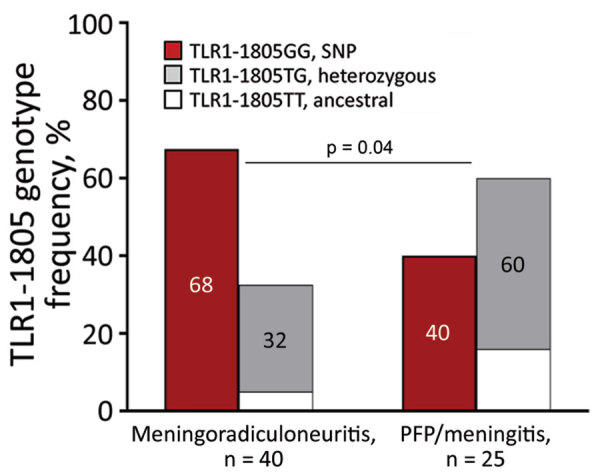
Frequency of TLR1–1805 genotypes, stratified by clinical manifestations (meningoradiculoneuritis and PFP/meningitis) among patients with Lyme neuroborreliosis treated in Ljubljana, Slovenia, during 2006–2013. The frequency of TLR1–1805 genotypes was determined via PCR restriction fragment-length polymorphism. Statistical analyses were performed using Fisher exact test for categorical variables or odds ratios. PFP, peripheral facial palsy; SNP, single-nucleotide polymorphism.

We previously demonstrated that the TLR1–1805GG SNP is associated with excessive immune responses, more symptomatic early infection, and a heightened risk for chronic inflammatory (antibiotic-refractory) Lyme arthritis, thereby linking host genetic variation with dysregulated immunity and severe Lyme borreliosis (*12*). Herein, we extended these findings to LNB (Figures 5, 6). As reported previously (*12*), 49% of the general population of Europe had both copies of the SNP (GG); distribution was similar in female and male patients. Similarly, 51% of patients with EM had both copies of the SNP, whereas the frequency was slightly higher in patients with LNB (57%), but these differences were not statistically significant (Figure 5). However, when we subcategorized LNB patients by clinical presentation, patients with meningoradiculoneuritis had a significantly higher frequency of TLR1–1805GG SNP compared to patients with PFP/meningitis (68% vs. 40%; odds ratio 3.1; p = 0.04) (Figure 6). Consent for genetic studies was not obtained from patients with suspected LNB, and they were not included in these analyses.

## Discussion

CNS involvement is a common manifestation of Lyme borreliosis in Europe. Although distinct manifestations of LNB are well described clinically, the reasons for these differences are not well understood. Herein, we demonstrated that LNB patients with meningoradiculoneuritis have a different immune signature than LNB patients without radiculoneuritis. Although both LNB groups (patients with meningoradiculoneuritis and PFP/meningitis) have broadly elevated innate and adaptive inflammatory immune response that the suspected LNB group does not, we observed the most dramatic differences for cytokines and chemokines associated with adaptive T- and B-cell immune responses in CSF. In particular, patients with meningoradiculoneuritis had the highest CSF levels of B-cell chemoattractants CXCL12 and CXCL13, as well as T-cell chemoattractants CXCL9 and CXCL10, and a potent Th17 cytokine, IL-17. These inflammatory mediators were often concentrated 10- to 50-fold in CSF, the site of the disease, and correlated strongly with lymphocyte counts (p<0.001), implying a role in CNS inflammation and disease pathogenesis. In contrast, cytokine and chemokine levels did not correlate with symptom duration, implying that greater immune responses are not caused simply by the longer disease duration in patients with meningoradiculoneuritis. Rather, we postulate that these site-specific T- and B-cell responses likely play a direct role in the pathophysiology of meningoradiculoneuritis and the higher prevalence of systemic symptoms in this condition. T- and B-cell mediators, namely IL-17 and CXCL13, have been implicated in disease pathogenesis in a recent study of patients with LNB or suspected LNB in Sweden (*32*), although that study did not compare patients with distinct manifestations of LNB. In contrast to findings for CSF, we observed low levels of most of the immune mediators in serum in LNB patients that were comparable to the levels in healthy persons (data not shown). Collectively, these findings support the role of site-specific T- and B-cell responses in the pathogenesis of borrelial meningoradiculoneuritis.

Because the clinical manifestation of LNB is largely attributable to host immune responses, immune mediators in CSF offer attractive targets as biomarkers for diagnosis as well as markers of disease activity and resolution. LNB diagnosis is limited because CSF pleocytosis and clinical signs and symptoms of disease overlap with other neurologic conditions and are thus not unique to LNB. Moreover, borrelia-IT-Abs, although highly specific, take weeks to develop and thus offer limited sensitivity during early infection. In addition, once elevated, antibodies against borrelia can persist for years and may not be able to distinguish previous from current infection. Immune mediators such as CXCL13, which are presumably elevated before demonstratable cellular influx necessary for CSF pleocytosis and borrelia-IT-Ab-S, and decrease dramatically after antimicrobial therapy, appear to address these diagnostic limitations (*20*). Indeed, CXCL13 has been proposed as an adjunct diagnostic marker for LNB (*19*–*26*). However, studies in the United States and Europe have demonstrated that the diagnostic utility of CXCL13 is primarily limited to patients with definitive LNB who already meet the standard diagnostic criteria (CSF pleocytosis, *Borrelia* IT-*Bb-*Ab-S) (*18*,*33*,*34*). Our findings underscore this point by demonstrating that CSF levels of CXCL13 are below the proposed 162 pg/mL diagnostic cutoff (*26*) in most patients with suspected LNB (median CXCL13 levels 1 pg/mL) and even in many patients with PFP/meningitis (median CXCL13 levels 53 pg/mL). Our results suggest that CXCL13 diagnostic utility may be constrained to patients with meningoradiculoneuritis, a clinically identifiable condition. Nevertheless, because of their role in pathogenesis, immune mediators remain attractive targets as biomarkers, and new cytokines with greater discriminatory power than CXCL13 have been proposed in LNB (*16*). As we have demonstrated, diagnostic suitability of these factors will require detailed studies of LNB patients with a range of well-defined clinical manifestations.

In addition to elevated immune responses, patients with borrelial meningoradiculoneuritis had a greater frequency and longer duration of constitutional symptoms than patients without radiculoneuritis. The most prevalent symptoms were sleep disturbance/insomnia, followed by fatigue, concentration disturbance, and memory disturbance, all of which could be attributed to sleeplessness resulting from pronounced radicular pain. However, these patients also had several other symptoms, such as low back pain, arthralgia, myalgia, and paresthesia, that are likely to have other causes, unrelated to sleeplessness. In contrast, patients with suspected LNB more often sought care for nonspecific symptoms such as headache, nausea, malaise, and vertigo. Thus, meningoradiculoneuritis is a distinct clinical entity distinguished not only by radicular pain but also by prevalence of several constitutional symptoms.

Alterations in the human genome have been implicated in maladaptive immune responses and severe Lyme borreliosis (*9*–*12*). We previously demonstrated that a SNP (1805GG) in TLR1, a major sensor for *Borrelia*, alters host immune responses to infection and thereby the clinical course and outcome of Lyme borreliosis (*12*). This SNP results in a transversion of thymine to guanine (1805T>1805G), causing an amino acid exchange of isoleucine for serine at position 602 in the transmembrane domain of the receptor, a constrained region likely to affect function. Indeed, the SNP results in decreased expression of TLR1 on cell surface and diminished downstream cytokine signaling in response to Pam_3_CSK_4_, a specific TLR1/2 agonist (*35*–*37*). Paradoxically, in response to *B. burgdorferi* infection, this SNP is associated with excessive immune responses with marked levels of IFN-γ and IFN-γ–inducible chemokines CXCL9 and CXCL10 in serum of patients with EM and even higher levels in joint fluid of those with postantibiotic (postinfectious) Lyme arthritis. This excessive inflammatory immune response was associated with highly symptomatic early infection in EM patients and with an elevated risk for postantibiotic chronic inflammatory Lyme arthritis (*12*). In this study, we extended these concepts to patients with various manifestations of LNB. We show that patients with meningoradiculoneuritis have a significantly higher frequency of the TLR1–1805GG SNP (OR = 3.1; p = 0.03) and more symptoms (p<0.001) than those with other LNB manifestations. Moreover, patients with meningoradiculoneuritis also have heightened T-cell (namely Th1) and B-cell immune responses, consistent with the role of this SNP in excessive Th1 inflammation and more symptomatic disease in patients with EM or Lyme arthritis.

A limitation of this work is that, because genetic studies require separate consent, the TLR1–1805GG analyses were performed in a different cohort of patients than those in whom the immune responses were tested; thus, we could not directly correlate the SNP genotype with the immune phenotype. Moreover, we based our study on a relatively small number of patients in each group. However, we should point out that this is an initial analysis of these concepts in LNB, and although small, these patient cohorts are well defined and are among the largest available. Finally, although the immune responses were elevated in CSF in patients with meningoradiculoneuritis as a group compared with those with PFP/meningitis, the levels of immune mediators varied in individual patients, and thus not every patient with radiculitis had higher CSF immune responses than did every patient with PFP/meningitis. Despite these limitations, these data support a link between TLR1–1805GG SNP, maladaptive Th1 immunity, and disadvantageous clinical outcomes in several manifestations of Lyme borreliosis, including LNB. An effort is now underway to recruit additional patients to validate these genetic and immunologic results.

In summary, our study demonstrates that meningoradiculoneuritis is a distinct clinical entity with unique immune and genetic pathophysiology. In future studies, we hope to establish the functional link between the TLR1–1805GG SNP, excessive site-specific T- and B-cell immune responses in CSF, and painful meningoradiculoneuritis. The findings from this study provide new considerations for the study of patients with meningoradiculoneuritis and LNB in general; further studies may help to improve accuracy of laboratory diagnosis.
